# The Kinase CIPK11 Functions as a Negative Regulator in Drought Stress Response in Arabidopsis

**DOI:** 10.3390/ijms20102422

**Published:** 2019-05-16

**Authors:** Yanlin Ma, Jing Cao, Qiaoqiao Chen, Jiahan He, Zhibin Liu, Jianmei Wang, Xufeng Li, Yi Yang

**Affiliations:** Key Laboratory of Bio-Resources and Eco-Environment of Ministry of Education, College of Life Sciences, Sichuan University, Chengdu 610065, China; maylok@163.com (Y.M.); Caojing94@163.com (J.C.); chenqqg7@sina.com (Q.C.); SCU_hjh@163.com (J.H.); lzb2003@163.com (Z.L.); wangjianmei@scu.edu.cn (J.W.); lixufeng0507@gmail.com (X.L.)

**Keywords:** *Arabidopsis thaliana*, drought stress, transcription factor, abiotic stress

## Abstract

Drought is a major limiting factor for plant growth and crop productivity. Many Calcineurin B-like interacting protein kinases (CIPKs) play crucial roles in plant adaptation to environmental stresses. It is particularly essential to find the phosphorylation targets of CIPKs and to study the underlying molecular mechanisms. In this study, we demonstrate that CIPK11 acts as a novel component to modulate drought stress in plants. The overexpression of *CIPK11* (*CIPK11*OE) in Arabidopsis resulted in the decreased tolerance of plant to drought stress. When compared to wild type plants, *CIPK11*OE plants exhibited higher leaf water loss and higher content of reactive oxygen species (ROS) after drought treatment. Additionally, a yeast two hybrid screening assay by using CIPK11 as a bait captures Di19-3, a Cys2/His2-type zinc-finger transcription factor that is involved in drought stress, as a new interactor of CIPK11. Biochemical analysis revealed that CIPK11 interacted with Di19-3 in vivo and it was capable of phosphorylating Di19-3 *in vitro*. Genetic studies revealed that the function of CIPK11 in regulating drought stress was dependent on Di19-3. The transcripts of stress responsive genes, such as *RAB18*, *RD29A*, *RD29B*, and *DREB2A* were down-regulated in the *CIPK11*OE plants. Whereas overexpression of *CIPK11* in *di19-3* mutant background, expression levels of those marker genes were not significantly altered. Taken together, our results demonstrate that CIPK11 partly mediates the drought stress response by regulating the transcription factor Di19-3.

## 1. Introduction

Osmotic stress that is imposed by soil salinity and drought is the main environmental problems around the world, which could lead toward extensive production losses in agriculture. Therefore, understanding how plants sense stress signals and adapt to adverse environments is critical in improving stress resistance in crops to achieve agricultural sustainability [1]. For drought stress, the primary signal for plant cells is related to osmotic stress [1]. To date, hundreds of genes that are involved in plant responses to drought stress have been identified, some are related to stress signal perception [2,3] and some are related to signal transduction and adaption [4,5]. When suffered from drought stress, the balance of plant cellular redox is altered, such as accumulation of reactive oxygen species (ROS), which affects normal growth and development [6]. Plants recruit positive and negative regulators of drought stress for fine-tuning the balance between the growth and stress responses. In recent years, some key components, including both negative and positive regulators, have been identified to be involved in plant responses to abiotic stresses.

Calcium (Ca^2+^), as an important second messenger in plants, is stimulated by various environmental changes, such as drought and salt, and is responsible for transducing the signals to downstream components in specific signal transduction pathways [7]. The calcineurin B-like proteins (CBLs) work as primary component in Ca^2+^ sensors that sense Ca^2+^ signal and interact with and activate a family of Ser/Thr protein kinases, called CBL interacting protein kinases (CIPKs), which modulate a large array of cellular processes [8].

So far, there are 10 CBLs and 26 CIPKs that are found in Arabidopsis and part of CBL-CIPK pathways are widely characterized in recent years [9,10]. The first revealed a CBL-CIPK working pair is the SOS system, CBL4 and CIPK14, also known as SOS3 and SOS2, function as positive regulators in salt stress through activating the plasma membrane (PM)-located Na^+^/H^+^ exchanger SOS1 in Arabidopsis [11,12]. The CBL1/9-CIPK23 complex functions in multiple signal responses and the regulatory mechanisms are deeply studied. When the plants suffer from low potassium stress, CBL1/9-CIPK23 complex phosphorylates and actives potassium transporter HAK5 and AKT1 to facilitate potassium uptake [13]. The CBL1/9-CIPK23 pathway also participates in ABA-mediated stomatal movement by modulation of annion channel SLAC1 and SLAH3 [14]. Other CBL-CIPK complexes, such as CBL4-CIPK6 and CBL2/3-CIPK21, are also identified as important components in stress signal transduction pathways [15,16]. Several transcription factors have been identified to be downstream targets of the CBL-CIPK complex, the Ethylene Responsive Factor 7 (ERF7), a transcriptional repressor of ABA signaling, was reported to be regulated by CIPK15 [17]. CIPK11 regulates the ABA insensitive 5 (ABI5), which is involved in the regulation of ABA response [18]. However, downstream components of most CIPKs and the regulating mechanisms under multiple abiotic and biotic stress conditions still need to be uncovered.

The Di19 (dehydration-induced19) protein family is a small transcription factor family that consists of seven hydrophilic proteins, which contain two hydrophilic Cys-2/His-2 zinc-finger-like domains, and were reported to be involved in abiotic stress [19,20]. Most of the family members are ubiquitously expressed in all organs and predominantly localize in the nucleus [19]. In Arabidopsis, the first reported Di19 protein was Di19-1, which participated in response to drought stress by binding to the TACA(A/G)T elements in the pathogenesis-related *PR1*, *PR2*, and *PR5* promoters and positively regulating their expressions [19,20]. Recently, Di19-1 was also found to interact with polycomb-group repressor MEDEA (MEA) and recruit it at the *RESISTANCE TO P. SYRINGAE 2* (*RPS2*) promoter to negatively regulate the immune response [21]. Di19-3, as a transcriptional activator, was involved in plant response to high salinity, drought, ABA, and H_2_O_2_ [22]. Although multiple functions of Di19-3 have been reported, its upstream regulators are still largely unknown. Interestingly, Di19-7 functions in regulating light signaling and abiotic responses, but abiotic treatments do not affect its expression [23]. Most Di19 family proteins interacted with CDPKs (calcium-dependent protein kinases) and were thereby strongly phosphorylated by CDPKs in vitro in a Ca^2+^-dependent manner [19,24]. In other species, the overexpression of *GhDi19-1* and *GhDi19-2* in Arabidopsis resulted in the increased sensitivity to high salinity and exogenous ABA [25]. Recently, a study reported that OsDi19-4 acts downstream of OsCDPK14 to positively regulate the ABA response by modulating the expression of ABA-responsive genes in rice [26]. These studies suggest that Di19 family proteins functionally participate in different abiotic signaling pathways as well as responses to pathogenic stress.

Information from CBL-CIPK has enriched the core knowledge of the plant response to diverse stress. However, different downstream targets and the mechanisms that most of CIPKs participate in abiotic and biotic stress responses still need to be studied. In this study, we reported that *CIPK11* overexpression plants (*CIPK11*OE) exhibited hypersensitivity to drought stress by increased water loss from leaves and enhanced ROS accumulation. Besides, we also found that CIPK11 interacts with and phosphorylates the transcription factor Di19-3. Plants lacking functional Di19-3 showed greater drought tolerance, while the overexpression of *CIPK11* in *di19-3* mutant background could not rescue the drought tolerant phenotype of the *di19-3* mutant. These data suggest that CIPK11 plays a role in drought stress response, at least in part, through its interaction with, and phosphorylation of, transcription factor Di19-3.

## 2. Results

### 2.1. Expression of CIPK11 is Induced by Drought

Previously, RNA gel blot analysis indicated that the expression of *CIPK11* has been shown to be induced by drought [27]. To investigate the possible function of the kinase CIPK11 in the drought response, the transcriptional abundance of *CIPK11* was analyzed by using the Electronic Fluorescent Pictograph (e-FP) browser [28]. Based on the microarray data from the AtGenExpress Visualization Tool, the *CIPK11* transcripts were upregulated 3.0 folds in rosette after treatment with drought (rafts were exposed to the air stream for 15 min. with loss of apparently 10% fresh weight) for 0.5 h and 3.24 folds in rosette after treatment with drought for 1 h (Figure S1). The quantitative reverse transcription-polymerase chain reaction (qRT-PCR) assay showed that the *CIPK11* transcripts were up regulated 1.75 folds in rosette leaves under drought stress treatment for seven days in wild-type plants (Col-0), which is consistent with the microarray data (Figure S2). These results provide the possibility that CIPK11 may be involved in drought stress response.

### 2.2. Overexpression of CIPK11 Confers Drought Hypersensitivity

**Figure 1:**
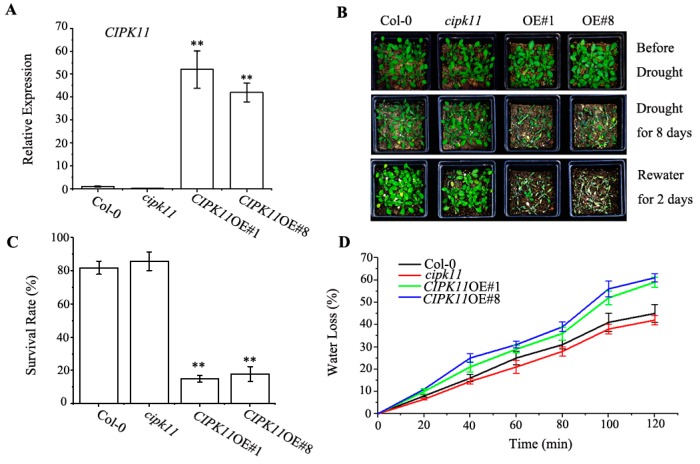
The *CIPK11*-Overexpression Plants Are Hypersensitive to Drought Stress. (**A**) Quantitative measurement of the *CIPK11* transcript levels in 28-day-old Col-0, *cipk11*, *CIPK11*OE#1 and *CIPK11*OE#8. Values are means ± SD (*n* = 3). (**B**) Drought-resistance assay. Images showing the drought stress phenotypes of twenty-eight-day-old Col-0, *cipk11*, *CIPK11*OE#1, and *CIPK11*OE#8 plants before drought (top), drought stress for 10 days (middle), and two days after re-watering (bottom). (**C**) The survival rates of Col-0, *cipk11*, *CIPK11*OE#1, and *CIPK11*OE#8 plants after 10 days of drought stress and then four days of re-watering was determined. Values are means ± SD (*n* =5 biological replicates, 16 plants in each replicate). (**D**) Water loss in Col-0, *cipk11*, *CIPK11*OE#1, and *CIPK11*OE#8 plants leaves (*n* = 3 biological replicates, each replicate consisting of three fully expanded leaves from five-week-old plants subjected to drought stress for four days). y** *p* < 0.01; Student’s *t*-test.

To investigate whether CIPK11 modulates the drought stress response in plants, T-DNA insertion mutant of *CIPK11* (*cipk11*) was obtained from the Arabidopsis Biological Resource Center (ABRC), and *cipk11* homozygous lines were isolated by RT-PCR. The *cipk11* mutant (Salk_108074) has a T-DNA insertion in the 456 bp downstream of the predicted ATG start site of *CIPK11* (Figure S3). The qRT-PCR analysis showed that the expression level of *CIPK11* was completely disrupted in the *cipk11* mutant (Figure 1A). Furtherly, *CIPK11* over-expression (*CIPK11*OE) plants was generated by transforming with CaMV 35S promoter-driven *CIPK11* tagged with a C-terminal *Flag* epitope in Col-0 plants to investigate its potential function during drought stress. In all, we obtained 12 independent T_3_ homozygous lines and verified them via PCR analysis (data not shown). Two representative lines (*CIPK11*OE#1 and *CIPK11*OE#8) with the highest expression were selected for further examination. The results of qRT-PCR showed that the transcripts of *CIPK11* were increased 52 times in *CIPK11*OE#1 and 43 times in *CIPK11*OE#8 when compared with Col-0 (Figure 1A).

When subjected to drought condition for 8 d, the *CIPK11*OE plants were extremely sensitive to drought stress, while the *cipk11* mutant plants exhibited slight drought tolerant phenotype as compared with the Col-0 (Figure 1B). After re-watering for 2 d, the *CIPK11*OE plants almost could not recover normal growth, while the *cipk11* mutant plants did not show any observable difference when compared to wild type plants, both of them recovered normal growth (Figure 1B), suggesting that there are functionally redundant proteins with CIPK11, which may be involved in response to drought stress of plants. The analysis of the survival rate demonstrated that only 15% of *CIPK11*OE#1 and 18% of *CIPK11*OE#8 survived as compared with over 82% of Col-0 and 86% of *cipk11* mutant plants after a 8-d period of drought stress treatment and a 2-d period of re-watering (Figure 1C). Additionally, the analysis of water loss rates revealed that the *CIPK11*OE#1 and *CIPK11*OE#8 lost 62.5% and 60% of their initial fresh weight compared to 45% in *cipk11* mutant and 41% in Col-0 plants after leaves were detached for 2 h on open Petri dishes (Figure 1D), indicating that water loss of detached rosette leaves of *CIPK11*OE plants was much faster than Col-0 plants and *cipk11* mutants. These results revealed that overexpression of *CIPK11* decreased the resistance to dehydration stress in Arabidopsis. Interestingly, *CIPK11* was strongly induced by drought stress at the transcriptional level, suggesting that CIPK11 may play an important role in balancing plant growth and stress response.

### 2.3. Physiological Properties in CIPK11-Overexpressing Arabidopsis under Drought Treatment

**Figure 2:**
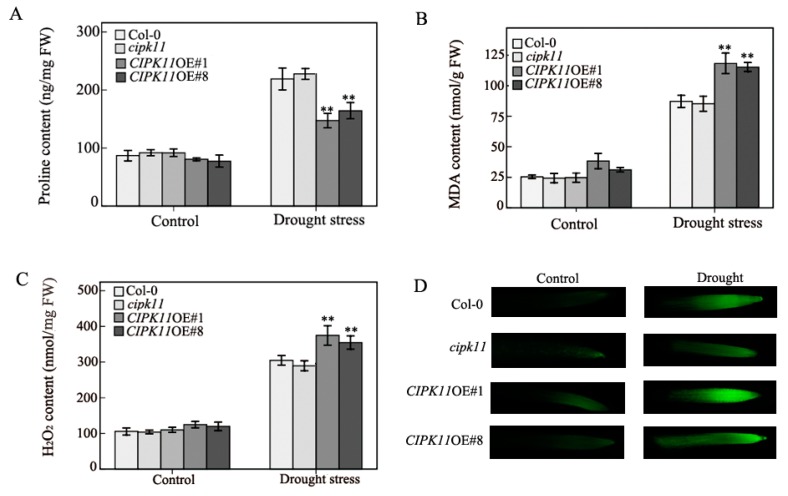
The overexpression of *CIPK11* increases cell damage caused by drought stress (**A**–**C**) The relative Proline, MDA, and H_2_O_2_ level in the Col-0, *cipk11*, *CIPK11*OE#1, and *CIPK11*OE#8 were calculated under normal and drought stress conditions. Error bars indicate SE (*n* = 3) and asterisks indicate significant differences from the Col-0 using the unpaired Student’s t-test (* *p* < 0.05; ** *p* < 0.001). (**D**) Detection of ROS by using H_2_DCFDA. Roots from Col-0, *cipk11*, *CIPK11*OE#1, and *CIPK11*OE#8 seedlings were subjected to liquid MS medium (Control) and exposed in room air for three minutes to simulate drought stress. The fluorescence of ROS (green) was imaged in the cells of root and the root images were collected after 15 min. incubation with H_2_DCFDA. Fluorescence indicates the presence of ROS.

Analyses of physiological indices, such as proline, H_2_O_2_, and MDA content, are often used to assess the stress tolerance in plants. Proline is considered to be an important osmotic adjustment substance by avoiding dehydration in many species under abiotic stress. To investigate the differences in stress tolerance between *CIPK11* overexpression and wild type plants at the physiological level, we tested the contents of proline, under normal and drought conditions. Our results demonstrated that the proline contents in *CIPK11*-overexpressing Arabidopsis lines showed no significant difference to Col-0 under normal condition. However, when the adult plants were grown under drought condition, the proline contents of *CIPK11* over-expression plants were much lower than those of wild type plants, which suggests that the over-expression of *CIPK11* increases plant sensitivity to drought stress (Figure 2A). Drought stresses also lead to a rapid and excessive accumulation of cell-damaging ROS species in plant cells, and the content of MDA and H_2_O_2_ are usually used to represent a reliable indicator for the degree of ROS-caused membrane oxidative damage under various stress conditions. Our findings revealed that there was no difference in MDA and H_2_O_2_ production without drought stress among all of the plants tested, whereas the MDA and H_2_O_2_ levels in the leaves of transgenic plants over-expressing *CIPK11* were higher than in Col-0 at 21 d after drought treatment, indicating that *CIPK11*OE plants suffered much cell injury than the Col-0 and *cipk11* plants (Figure 2B,C). In addition, we used the fluorescent dyes H_2_DCFDA to investigate the production of ROS in the root cells of Col-0, *cipk11*, *CIPK11*OE#1, and *CIPK11*OE#8 with the laser scanning confocal microscopy. The plants tested were exposed in room air for three minutes to simulate drought stress for studying the physiological response in root of seedlings. Our results showed that the fluorescence intensity in the root cells of *CIPK11* over-expression lines were dramatically increased when compared with Col-0 and *cipk11* plants under drought stress condition (Figure 2D). These results were consistent with the lower survival rate of *CIPK11*OE plants after drought stress treatment. Taken together, our results showed that the kinase CIPK11 functions as a general and negative regulator in drought stress resistance through affecting the contents of various stress-related chemical compounds, including proline, MDA, and H_2_O_2_ under dehydration stress condition.

### 2.4. Identification of Di19-3 as A CIPK11-Interacting Protein

We next undertook the identification of CIPK11-interacting protein(s) to gain insight into the function of CIPK11 in drought stress. In this study, a yeast two-hybrid assay was performed by using the full-length of CIPK11 as bait to screen its interacting protein. Indeed, a series of putative CIPK11-interacting proteins were identified (data not shown), among them, a transcription factor that was encoded by AT3G05700 (Di19-3) was captured as a potential CIPK11-interacting protein.

**Figure 3:**
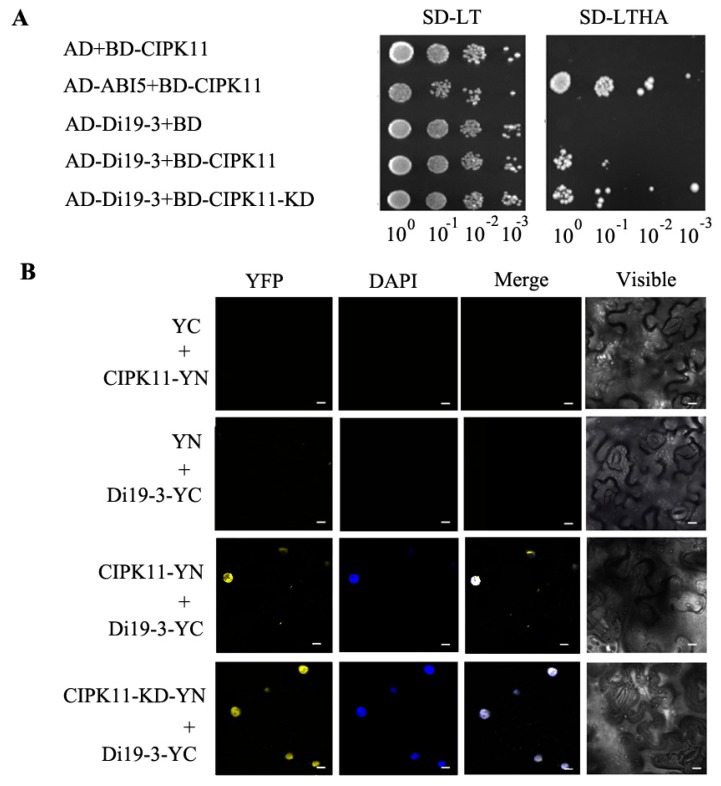
CIPK11 interacts with dehydration-induced19-3 (Di19-3). (**A**) Interaction between Di19-3 and CIPK11 was further confirmed by yeast-two-hybrid assay. Interaction was indicated by growth on selection medium (SD-LTHA) (right). Growth on SD-LT was used as control (left). SD-LT, SD/Trp/Leu media; SD-LTHA, SD/Trp/Leu/His/Ade media. (**B**) CIPK11 interacts with Di19-3 in vivo. Co-transformation of CIPK11-YN and Di19-3-YC led to the reconstitution of YFP signal, CIPK11-KD-YN and Di19-3-YC also led to the reconstitution of YFP signal whereas no signal was detected when CIPK11-YN and YC or Di19-3-YC and YN were co-expressed. The images were obtained from the yellow fluorescent protein channel or bright field, or a merged picture of the two. YFP images, DAPI stained images, visible light images, and merged images were taken. Scale bar = 10 μm. The experiments were performed twice with similar results.

To confirm the specificity of CIPK11 interaction with Di19-3, we carried out the yeast two-hybrid assay to further study the interaction between them. Our results revealed that the yeast cells co-transformed with AD-Di19-3 and BD-CIPK11/CIPK11KD (KD is referred as kinase domain), as well as AD-ABI5 and BD-CIPK11, a positive control, grew well on Trp/Leu/His/Ade-deficient medium, while the growth of other yeast cells was completely inhibited (Figure 3A), which suggests that both full-length CIPK11 and CIPK11KD are able to directly interact with Di19-3. The analysis of subcellular localization revealed that CIPK11 was localized in the cytoplasm and nucleus in epidermal cells of tobacco leaves, which was consistent with that previously reported [18], while Di19-3 was only localized in the nucleus (Figure S4). To monitor the interaction between CIPK11 and Di19-3 in plant cells, the full-length *CIPK11* or *CIPK11-KD* was introduced into the plant expression vector pUC-SPYNE and *Di19-3* was introduced into the pUC-SPYCE vector to perform the bimolecular fluorescence complementation (BiFC) assay. The result showed that the Yellow Flourescent Protein (YFP) signal was only detected in the nucleus of CIPK11-YFPN/Di19-3-YFPC-cotransfected tobacco leaf epidermal cells, which was coincident with DAPI staining, while the YFP signal was not detected with any single construct that was expressed individually or when CIPK11-YFPN and pUC-SPYCE or Di19-3-YFPC and pUC-SPYNE were expressed (Figure 3B), which suggests that CIPK11 specifically interacts with Di19-3 in the nucleus and the kinase domain of CIPK11 is required for this interaction.

### 2.5. CIPK11 Phosphorylates Di19-3 In Vitro

**Figure 4:**
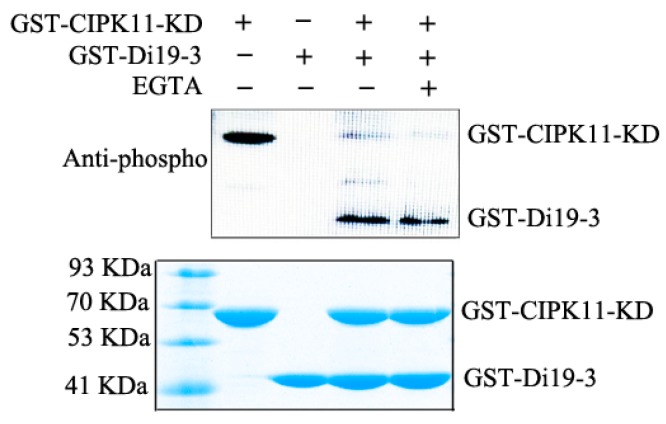
Phosphorylation of Di19-3 by CIPK11 *in vitro.* In vitro phosphorylation assay of Di19-3 was detected by immunoblotting with an anti-phosphothreonine antibody. The loading amounts of proteins were visualized with the Coomassie Brilliant Blue (CBB) stained gel (bottom panel). The experiment was conducted with at least three biological replicates.

Based on the evidence that CIPK11 directly interacts with Di19-3, we then wonder whether CIPK11 could phosphorylate Di19-3. To verify this hypothesis, we examined the ability of CIPK11 to phosphorylate Di19-3 using an in vitro kinase assay. GST-tagged CIPK11KD and Di19-3 were expressed in the *E. coli* system and the fusion proteins were purified using glutathione S-Sepharose 4B (GS4B, GE Healthcare, PA, USA) resin. After incubation of the purified proteins in kinase buffer, we separated the protein by SDS-PAGE and detected the phosphorylated proteins on the gel while using an anti-phosphothreonine antibody and visualized total proteins with CBB staining. As shown in Figure 3, an autophosphorylation band of ~60 kDa corresponding to GST-CIPK11-KD was observed in all of the samples, which suggests that purified GST-CIPK11-KD has the protein kinase activity. We also detected a phosphorylation band of ~47 kDa corresponding to GST-Di19-3 only at the presence of GST-CIPK11-KD fusion protein (Figure 4). The CBL–CIPK module is one of the pivotal players in transducing the Ca^2+^ signal in the plant cell. We assayed whether calcium has a direct effect on CIPK11-KD kinase activity by the addition of calcium ions and the calcium chelator EGTA to determine whether calcium is involved in this process. No difference in CIPK11-KD autophosphorylation or Di19-3 phosphorylation was seen in the presence of EGTA or calcium ions (Figure 4). These results indicate that the regulation of CIPK11-KD phosphorylate Di19-3 is calcium independent in vitro.

### 2.6. Genetic Interaction between CIPK11 and Di19-3 under Drought Stress

To investigate whether CIPK11 genetically interacts with Di19-3, a *Di19-3* T-DNA insertion line from TAIR (SALK_072390) was isolated. The location of the T-DNA insertion in *Di19-3* is shown in Supplemental Figure S5. It was previously reported that *Di19-3* loss-of-function mutants were tolerant to drought stress, whereas *Di19-3* overexpressing transgenic plants exhibited hypersensitive phenotype to drought condition [19]. We then crossed *CIPK11*OE#1 with the *di19-3* mutant to generate *CIPK11*OE#1/*di19-3* lines to further investigate the relationship between CIPK11 and Di19-3.

**Figure 5:**
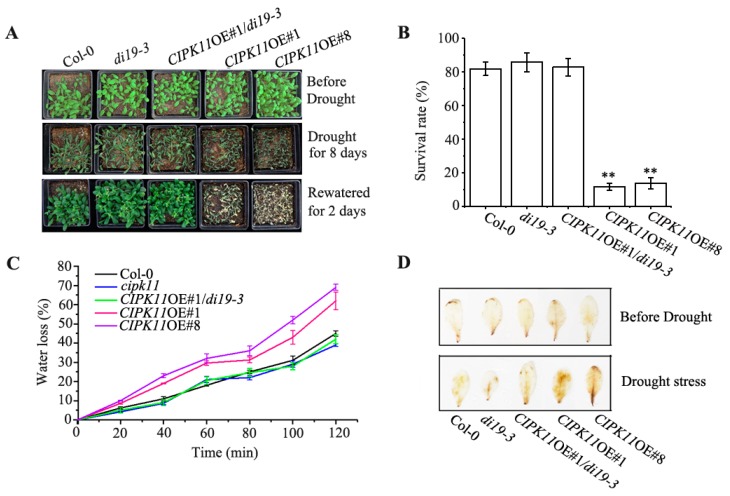
Genetic Interaction of CIPK11 with Di19-3 under drought stress. (**A**) Drought-resistance assay. Col-0, *di19-3*, *CIPK11*OE#1/*di19-3*, *CIPK11*OE#1, and *CIPK11*OE#8 plants grown under normal growth conditions for 28 days were subjected to drought stress condition for eight days and then re-watered for two days. (**B**) Survival rate after drought treatment for 10 days (*n* = 4 biological replicates, 16 plants in each replicate). Data represent means ± SD. The Asterisks above the columns indicate significant difference between means (** *p* < 0.01; Student’s *t*-test). (**C**) Water loss in Col-0, *di19-3*, *CIPK11*OE#1/*di19-3*, *CIPK11*OE#1, and *CIPK11*OE#8 leaves (*n* = 4, each replicate includes three fully expanded leaves from five-week-old plants grown in soil and subjected to drought for four days. Data represents means ± SD. (**D**) H_2_O_2_ content in Arabidopsis under drought treatment. DAB staining assay of rosette leaves of Col-0, *di19-3*, *CIPK11*OE#1/di19-3, *CIPK11*OE#1, and *CIPK11*OE#8 after drought stress.

When *CIPK11* was overexpressed in *di19-3* background, the transgenic plants were as tolerant to drought stress as the *di19-3* plants (Figure 5A), which further demonstrates that CIPK11 acts as an upstream regulator of Di19-3. As shown in Figure 5B, *CIPK11*OE plants did not survive under a 22-d period of drought stress treatment (the “severe” drought stress treatment), even after re-watering. However, the *CIPK11*OE#1/*di19-3* plants not only survived, but they also recovered well after re-watering compared with Col-0 plants. Consistent with these results, the water loss assays showed that the detached leaves of *CIPK11*OE#1/*di19-3* plants, similar to the Col-0, lost water lower than *CIPK11*OE plants under dehydration treatment. (Figure 5C). Additionally, the ROS content in all plants were analyzed by using DAB staining assay, as compared with Col-0 plants, the *CIPK11*OE plants accumulated more ROS, while both *di19-3* plants and *CIPK11*OE#1/*di19-3* lines accumulated less ROS under drought stress condition. (Figure 5D). These results strongly support an idea that indicates that CIPK11 and Di19-3 function in the same signaling pathway in plant responses to drought stress and the CIPK11 function is dependent of transcription factor Di19-3 during dehydration.

**Figure 6:**
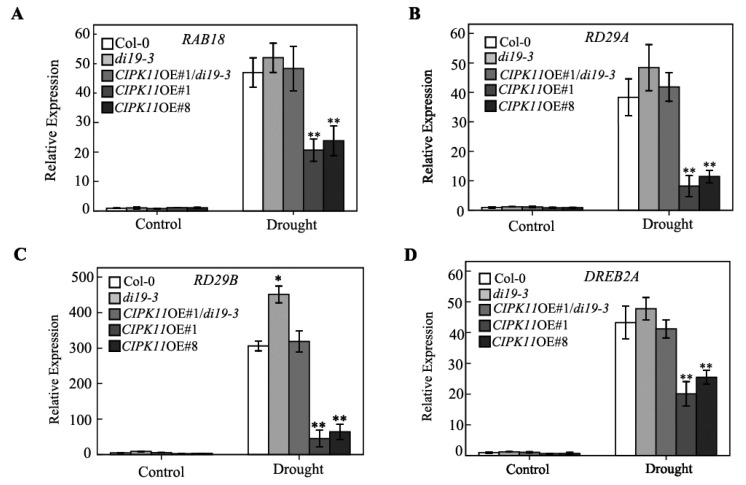
Expression profiles of the drought stress-responsive genes in Col-0, *di19-3*, *CIPK11*OE#1/*di19-3*, *CIPK11*OE#1, and *CIPK11*OE#8 in response to drought stress. Relative expression levels of the drought stress-responsive genes *RAB18* (**A**), *RD29A* (**B**), *RD29B* (**C**), and *DREB2A* (**D**) in Col-0, *di19-3*, *CIPK11*OE#1/*di19-3*, *CIPK11*OE#1, and *CIPK11*OE#8 plants under drought stress. Drought treatment was induced by withholding water for five days. The transcriptional levels were determined by qRT-PCR analysis. Values are the means ± SD (*n* = 3). The *Actin2* was used as an internal control. ** *p*<0.001, Student’s *t*-test.

The overexpression of *CIPK11* altered the expression pattern of stress-responsive genes in Arabidopsis. When considering that *CIPK11*OE plants decrease plant tolerance to drought, the transcript levels of several drought-induced marker genes may be different among the Col-0, mutants, and transgenic plants. To evaluate our hypothesis, the expression of several known drought-related genes, such as *RAB18*, *RD29A*, *RD29B*, and *DREB2A*, were analyzed under drought stress condition. Our results demonstrated that the expressions of all the marker genes tested were obviously down-regulated in *CIPK11*OE plants, but up-regulated in *di19-3* mutant plants, while their expressions in *CIPK11*OE#1/*di19-3* plants were not significantly altered when compared with the Col-0 plants under the drought condition (Figure 6). Taking together, our results strongly imply that the CIPK11-Di19-3 pathway participates in drought stress response by affecting a wide range of progresses, such as physiological alteration and drought-induced gene expressions.

## 3. Discussion

### 3.1. CIPK11 is A Negative Regulator for Plant Tolerance to Drought

The environmental stresses, such as drought and high salt, can initiate the Ca^2+^ signaling, which has a resultant impact on gene expression and cell physiology [29]. In recent years, the Ca^2+^-CBL-CIPK network that is involved in abiotic stress has been extensively studied [30,31,32,33]. However, their roles and underlying mechanisms are different. For example, Under salt stress, CIPK24 was activated by CBL4 and positively regulates SOS1 to mediate Na^+^/H^+^ exchange in plant roots [34]. Later studies have shown that the CBL10-CIPK24-SOS1 form a module by regulating Na^+^ detoxication in shoots and leaves to protect the shoot tissues from salt stress [35].

In contrast to our results for *CIPK11*, the overexpression of other *CBLs* or *CIPKs*, including *CBL1*, *CBL9*, *CIPK1*, and *CIPK6*, increased drought tolerance in Arabidopsis [36,37], demonstrating an indispensable role of the CBL-CIPK network in response to drought stress. Moreover, some CBL-CIPK modules of Arabidopsis have been unequivocally proven to exist in other species [38]. Some CIPKs from rice, maize, and cassava plants were found to participate in the response against drought stress [10,39]. The overexpression of the *HbCIPK2* in Arabidopsis resulted in salt and osmotic stress tolerance [40]. Another study showed that BrCIPK1 confers tolerance to drought in transgenic rice, because it increases the proline contents in plants [41]. Determining the responses of CIPK11 and other CIPKs to various stresses and a demonstration of the underlying mechanisms in future will be hoped to enrich the core knowledge base of this module. The expression analysis of *CIPK11* revealed that the transcripts of *CIPK11* was induced by drought stress, but the *CIPK11*OE plants were more sensitive to drought (Figure 1), which suggests that CIPK11 may play a key role in balancing plant normal growth and stress response. In this study, the *cipk11* mutant showed the same phenotype as wild-type plants under drought stress, although the H_2_O_2_ contents in *cipk11* mutants are slightly lower than Col-0 plants, suggesting that other CIPKs, even other protein kinases, such as CPKs and SnRKs, must be functionally redundant with CIPK11 in response to drought stress. Besides, qRT-PCR results also showed that some typical drought-inducible marker genes, such as *RAB18*, *RD29A*, *RD29B,* and *DREB2A* were also largely down-regulated in *CIPK11*OE plants under drought treatment (Figure 6). Furthermore, the overexpression of *CIPK11* could cause plant sensitivity to drought stress with higher leaf water loss and a higher content of ROS when compared with the Col-0 plants. These results suggested that CIPK11 is a general and negative regulator in the drought stress response.

### 3.2. How does CIPK11 Participate in Regulating Drought Response?

Previous research has shown that CIPK11 was located in the cytoplasm and nucleus in Arabidopsis. This dual localization in cells may be dynamic and appears to facilitate its functions in both early and delayed responses of plants to drought stress. The plant-specific Di19 transcription factors play a vital role in the response to multiple stresses. The Di19 family mainly consists of seven members, and most members in Di19 family are involved in drought stress, but the specific regulatory mechanism is different. In this work, we found that Di19-3 can be phosphorylated by CIPK11 and it participates in the response against drought stress. Further systematic studies are needed to determine whether CIPK11 phosphorylates and regulates the function of other members of the Di19 family.

### 3.3. Identification of a Downstream Target of CIPK11

The identification and characterization of the specific target (or substrate) of a CIPK is a key step to understand the functions of CIPKs in major signaling pathways in plants [33]. Some substrates of CIPKs have been identified in the last decade, revealing their precise roles in plant development and stress responses. For example, Arabidopsis CIPK15 phosphorylates an APETALA2/EREBP-type transcription factor, ERF7, in the ABA signaling pathway [42]. Arabidopsis CIPK11 induces the transcription of pathogenesis-related (PR) genes by directly interacting with and phosphorylate NPR1 [43]. In this study, we revealed that the specific interaction between CIPK11 and Di19-3 existed and the important roles of CIPK11-Di19-3 pathway mediated the drought stress response (Figure 3 and Figure 4). The *CIPK11*OE/*di19-3* plants showed similar drought stress insensitivity to the *di19-3* single mutants when compared with *CIPK11*OE plants, suggesting that CIPK11 modulating the drought stress response is dependent of Di19-3 (Figure 5 and Figure 6).

CIPK11 interacted with and phosphorylated Di19-3 by regulating the downstream target genes that are involved in drought stress. However, CIPK11 may also have other substrates to be involved in such a stress signaling pathway and it is unknown whether Di19-3 directly regulates the drought stress responsible genes that were tested in our study. Taken together, the CIPK11 kinase participates in the drought stress response at least partly by the regulation of Di19-3 via phosphorylation.

### 3.4. Possible Activation Mechanisms of CIPK11 by Upstream Regulators

The interactions between the CBLs and CIPKs are very complicated, with multiple CBL proteins being able to interact with multiple CIPK proteins to fulfill different functions of CIPKs in plants. However, the interactions between specific CBLs and CIPKs are also induced by a specific condition to transduce the perceived calcium signal and form the Ca^2+^-mediated CBL-CIPK network in response to various stimuli [9,33]. Previous studies indicated that CIPK11 interacted with CBL1, CBL2, CBL3, CBL4, CBL5, CBL9, and CBL10, illustrating a multi-function of CIPK11 in various signal transduction pathways [44,45,46]. Therefore, it is also interesting work to determine which CBL(s) may participate in activating CIPK11 in *planta* and act in the same functional context in the CIPK11-Di19-3 pathway under drought stress.

The regulatory domain of Arabidopsis CIPKs, including CIPK11, has the NAF/FISL motif for CBL binding and a protein–phosphatase interaction (PPI) domain for interaction with phosphatases [16]. Some CIPK members were found to interact with PP2Cs, such as ABI1, ABI2, or AHG3 (ABA-HYPERSENSITIVE GERMINATION3) [8,42,47,48] to block the kinase activity of CIPKs, which suggests that posttranslational modifications, such as phosphorylation and dephosphorylation of CIPK11 by unknown upstream regulators (i.e., kinase and phosphatases), may also regulate the kinase activity of CIPK11 in response to stresses condition. [49].

### 3.5. CIPK11-Di19-3 Regulatory Pathway may be a Potential Target in Agricultural Production

Drought is considered to be the major limiting factor of crop yield and quality all over the world; it is therefore very urgent to increase the drought tolerance of crops. The gene editing technology, such as the CRISPR-Cas9 system, is widely considered to be an efficient manner to do so. In recent years, increasing progresses of genome-edited plants with many excellent traits have been achieved. Our presented data demonstrate that the kinase CIPK11 interacts and phosphorylates transcription factor Di19-3 to participate in the response of drought stress. Although downstream genes that are directly regulated by Di19-3 still need to be uncovered, our findings provide a theoretical and practical basis for the use of CIPK11-Di19-3 working pair in molecular breeding to enhance the stress resistance in crops. It is worth focusing on two aspects to provide a theoretical basis for practical application: one is the identification of other CIPKs that may be functionally redundant with CIPK11 in drought response, so as to construct multiple *cipk* mutants to further uncover the molecular mechanism underlying the function of plant-specific calcium regulatory kinase CIPKs. The other is to find the genes that are directly modulated by transcription factor Di19-3, which may deeply help us to understand the drought-CIPK11-Di19-3 signal transduction pathway. Additionally, there must be homologous genes of *CIPK11* and *Di19-3* as well as similar pathways, like CIPK11-Di19-3 in crops, so it is possible that constructing drought resistant crops with non-GM (genetically modified) technology by using the CRISPR-Cas9 system. In all, CIPK11 and Di19-3 could be the promising candidates for the construction of stress resistance crops in the future.

## 4. Materials and Methods

### 4.1. Plant Growth

The *Arabidopsis thaliana* ecotype Columbia (Col-0) was used for over-expression and stress response analysis experiments in this study. The Arabidopsis Col-0 was used as a wild-type control in our experiment. The T-DNA insertion lines, including *di19-3* (SALK_072390), and *cipk11* (SALK_108074) were obtained from the ABRC (http://www.arabidopsis.org/abrc/). Arabidopsis seeds were surface-sterilized with 100% (*v*/*v*) ethanol for one minute and 75% (*v*/*v*) ethanol for five minutes, and then washed with sterile water eight times, and plated on 1/2 × Murashige and Skoog (MS) medium (containing 1 % sucrose, *w*/*v*) with 0.8 % (*w*/*v*) agar. After stratification at 4 °C in the dark for 3 d, the seeds were transferred to a growth chamber (16-h light/8-h dark cycle at 23 °C) until the flowering stage for plant transformation and generation of seeds. The chamber was controlled at 21–23 °C, 100 μmol photons m^−2^ s^−1^, 60% relative humidity, and 16 h light/8 h dark cycles.

### 4.2. Quantitative Real-Time PCR Analysis

For quantitative expression analysis by qPCR, the total RNAs were extracted from various samples that were harvested at different time points using the RNAiso Plus reagent from Takara (Otsu, Japan). The RNA was treated with RNase-free DNase I (TaKaRa) to remove genomic DNA. The concentration and purity of the extracted RNA were determined using a NanoDrop2000 spectrophotometer. Two micrograms of RNA were used for reverse transcription with PrimeScript RT-PCR reagent Kit, according to the manufacturer’s instructions. qRT-PCR was performed with SYBR Premix Ex Taq II from Takara (Takara Bio Inc., Otsu, Japan) and used a CFX96TM Real Time System (Bio-Rad, Hercules, CA, USA). The PCR cycling parameters were set, as follows: 95 °C for 3 min., 45 cycles of 5 s at 95 °C, 30 s at 60 °C, and a final melting curve of 65 to 95 °C with an increment of 0.2 °C. Primers used in this study are shown in Table S1. *Actin2* was used as the endogenous control to normalize the variance among samples. Relative expression values were calculated by employing the 2^−ΔΔ*C*t^ method [50].

### 4.3. Constructs and Generation of Transgenic Plants

To study the functions of CIPK11 and Di19-3 in Arabidopsis, the full-length coding sequence of *CIPK11* and *Di19-3* were amplified by PCR and inserted into the overexpression vector pBIB-BASTA-35S -FLAG. All of the transgenic plants were generated through a floral dip method [51]. The T1 transgenic seedlings were selected by using BASTA and each T_2_ plant was individually collected. The selected T_2_ plants were propagated and confirmed by quantitative qRT-PCR analysis.

For generation of the yeast expression vector, the full length *CIPK11/CIPK11-KD* and *Di19-3* were cloned into the yeast expression vector pGBKT7 and pGADT7, respectively. The CDS of *ABI5* was cloned into pGADT7 for use as positive control. For generation of the BiFC vectors, the coding region of *CIPK11* and *CIPK11-KD* were cloned into pSPYCE, resulting in CIPK11-YC, and the coding region of *Di19-3* was cloned into pSPYNE, resulting in Di19-3-YN. All of the primers used for construction are listed in Table S1.

### 4.4. Yeast Two-Hybrid Assay

To detect the interaction between CIPK11 and Di19-3, the constructs, including BD-CIPK11, BD-CIPK11-KD, and AD-Di19-3, as well as positive and negative control plasmids, were co-transformed into *Saccharomyces cerevisiae* AH109 cells using the lithium acetate method according to the manufacturer’s protocol (Clontech, Mountain View, CA, USA). The cotransformants were selected on synthetic complete medium lacking Trp and Leu (SD-L-W) at 30 °C before detecting the interaction. After growing for 3d, the successfully transformed colonies were inoculated with shaking in liquid SD-L-W and grown overnight at 30 °C. The overnight culture was washed three times in 0.9% NaCl and OD_600_ was determined at 0.8. For the interaction analysis, the positive transformants were selected to separately spot on double-dropout medium SD/L-W and quadruple-dropout medium (SD/-Trp/-Leu/-His/-Ade, SD-LWHA) by gradient dilution. Photographs were taken after four days incubation at 30 °C.

### 4.5. Monitoring ROS in the Root Cells under Drought Stress

The detection of ROS production in the roots was performed using fluorescent dye 2’,7’-dichlorofluorescein diacetate (H_2_DCFDA) (Sigma-Aldrich, St. Louis, MO, USA), as described previously [52]. The seven-day-old seedlings were placed on a weighing paper at 22 °C to dry. After being exposed in air for three minutes, the whole seedlings were incubated with 50 μM H_2_DCFDA (dissolved in MES-KCl buffer, KCl 50 mM, MES 10 mM, pH 5.5) for 15 min. in dark. Excess H_2_DCF-DA solution from the surface of roots was then removed by washing the plants twice for 2 min. with MES-KCl buffer. The fluorescence in root cells was detected by a confocal microscopy using an excitation wavelength of 488 nm, and the relative fluorescence intensity of at least eight seedlings per sample were analyzed using ImageJ software.

### 4.6. Determination of Drought Responsive Physiological Indices

The 21-day-old plant leaves that were pre-treated under normal and drought stress conditions were collected for proline, MDA, and H_2_O_2_ content analysis. Drought stress treatment experiments were repeated three times. The proline, malondialdehyde (MDA), and H_2_O_2_ contents were determined by the proline assay kit, MDA assay kit, and H_2_O_2_ assay kit (Jiancheng, China), according to the instructions, respectively.

### 4.7. Bimolecular Fluorescence Complementation Assay

BiFC assays were performed, as described previously [53]. In brief, all of the constructs were transformed into the Agrobacterium strain GV3101. An equal volume of Agrobacterium harboring CIPK11-YC, CIPK11-KD-YC, and Di19-3-YN was re-suspended in injection buffer (liquid MS medium containing 150 μM acetosyringone, 10 mM MgCl_2_, 10 mM MES, pH 5.7) and mixed to a final concentration of OD_600_ = 0.8. The Agrobacterium cells containing the indicated constructs were infiltrated into young leaves of N. benthamiana. Plants were grown at 23 °C and allowed to recover for 48 h, and then the fluorescence signals of YFP were observed and imaged via confocal fluorescence microscopy with excitation wavelength at 513 or 488 nm, and the emission wavelength at 527 or 507 nm. The nucleus was stained with DNA dye 4,6-diamidino-2-phenylindole (DAPI). The experiment was repeated three times, each time with three or four biological replicates.

### 4.8. Protein Purification and Protein Phosphorylation Assay

In vitro kinase assays were performed, as described previously [54], with slight modification. CIPK11-KD and Di19-3 were cloned into pGEX-6p-1 vector and then expressed as a glutathione S-transferase (GST) fusion protein. The recombinant proteins were expressed in the *Escherichia coli* BL21 (DE3) strain and then were purified using glutathione Sepharose-4B chromatography, respectively. The in vitro kinase assays were performed using approximately 2μg of GST-CIPK11-KD and 6 μg of GST-Di19-3 in a 20 μL reaction buffer containing 20 mM Tris-HCl pH 7.5, 10 mM MgCl_2_, 1 mM ethylene glycol tetraacetic acid (EGTA), 1.1 mM CaCl_2_, and 50 μm ATP; the reactions without Di19-3 were used as control. After incubation at 30 °C for 30 min., the reactions were terminated by the addition of 5 μL of 5× loading buffer and heated at 99 °C for 5 min. The reaction products were fractionated by 10% SDS-PAGE and then transferred to a polyvinylidene fluoride (PVDF) membrane. The anti-pThr antibody (Cell Signaling Technology, 9381S) (1:1000) was used for immunoblotting and the signal was visualized while using chemiluminescence (Bio-Rad).

### 4.9. Abiotic Stress Tolerance Assays

The Col-0, *cipk11*, *di19-3*, and the T_3_ generation of the two independent *CIPK11*OE transgenic lines were used to evaluate the tolerance for drought stress. The surface sterilized seeds of all lines were grown on mixed soil, as described above. For the phenotype assay under the drought stress, all of the plants were grown under normal watering, individual pots containing an equal weight of a dry soil/vermiculite (1:3, *v*/*v*) mixture in a controlled culture room. The four-week-old Arabidopsis plants were deprived of water for eight days and then recovered for two days. In addition, four-week-old Arabidopsis leaves were used for the water loss rate assay. Leaves of similar size and positions on each kind of plants were trimmed and weighed for water loss when compared with the initial fresh leaves. The survival rate of all lines was scored based on the observation of actively re-growing seedlings as survivors and nongrowing or wilted seedlings as non-survivors after drought stress treatment. The plants were photographed and the survival rates were investigated until the wild-type or mutant leaves showed primary wilting. Each experiment was repeated at least three times, and the student’s t-test statistically analyzed the data.

### 4.10. DAB Staining

H_2_O_2_ was detected by DAB staining, as described previously [55]. Three fully expanded leaves from each drought treated Arabidopsis plant were excised and placed in a microtiter plate that was supplemented with 2 mL DAB staining solution (1 mg/mL DAB, Sigma-Aldrich, adjust to pH 3.8 with HCl), then vacuumed for 5 min. After 5 h incubation with gentle shake, the leaves were immersed in boiling 80% (*v*/*v*) ethanol for 10 min. to terminate the staining, and the leaves were boiled in the bleaching solution (ethanol, acetic acid, glycerol= 3:1:1) for 15 min. to decolor the leaves (except for the deep brown poly-merization product that was produced by the reaction of DAB with H_2_O_2_) and then the image was captured.

### 4.11. Statistical Analysis

The data are represented as means ± SD. Statistical analysis was performed using Student’s t-test, with the use of SPSS Software, Version 16.0. Values of *p* < 0.05 were considered to be significant, and Values of *p* < 0.01 were considered as more significant. Analysis of variance was used to compare the significant difference based on Student’s *t*-test (*n* = 4).

## Supplementary Materials

Supplementary materials can be found at https://www.mdpi.com/1422-0067/20/10/2422/s1.
