# Supplementary material for: The Kinase CIPK11 Functions as a Negative Regulator in Drought Stress Response in Arabidopsis
Source: Int J Mol Sci. 2019 May 16;20(10):2422. doi: 10.3390/ijms20102422 (PMC6566343; doi:10.3390/ijms20102422)
Supplement: Supplementary file 1 [file ijms-20-02422-s001.pdf]

International Journal of *Molecular Sciences*

MDPI

Supplementary

The kinase CIPK11 functions as a negative regulator in drought stress response in Arabidopsis

Yanlin Ma, Jing Cao, Qiaoqiao Chen, Jiahan He, Zhibin Liu, Jianmei Wang, Xufeng Li and Yi Yang*

Key Laboratory of Bio-Resources and Eco-Environment of Ministry of Education, College of Life Sciences, Sichuan University, Chengdu 610065, China; maylok@163.com (Y.M.); Caojing94@163.com (J.C.); chenqqg7@sina.com (Q.C.); SCU_hjh@163.com (J.H.); lzb2003@163.com (Z.L.); wangjianmei@scu.edu.cn (J.W.); lixufeng0507@gmail.com (X.L.)

* Correspondence: yangyi528@scu.edu.cn

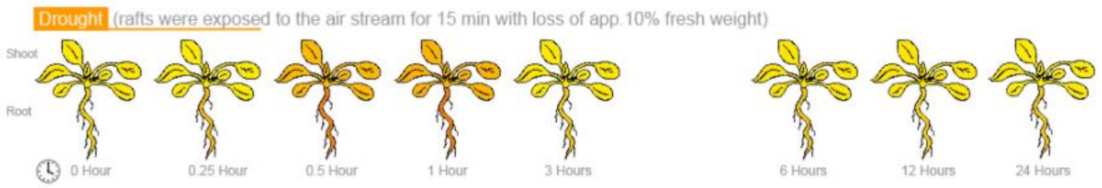

Figure S1. Inducible expression of *CIPK11* by drought stress obtained from the Electronic Fluorescent Pictograph (e-FP) browser.

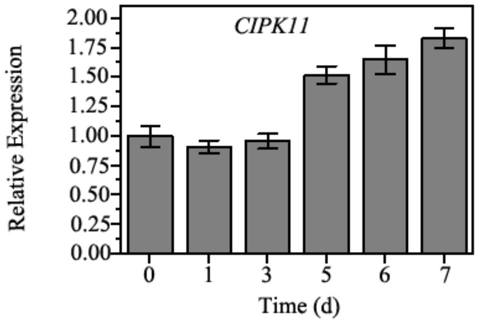

Figure S2. Quantitative measurement of the *CIPK11* transcript levels in 28-day-old Col-0 plants after drought stress for 0, 1, 3, 5, 6, or 7 days for qRT-PCR. Actin 2 was used as internal control. Values are means ±SD (n = 3).

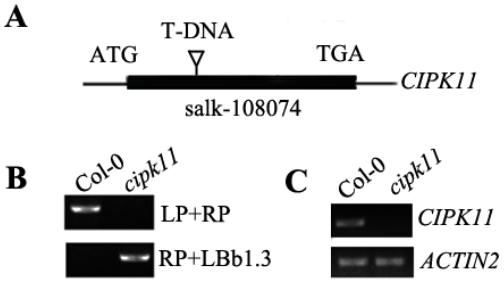

Figure S3. Identification of Arabidopsis *cipk11* mutant. (A) Structure of Arabidopsis *CIPK11* and T-DNA insertion site in *cipk11* mutant (Salk_108074). (B) Molecular analysis of *cipk11* and Col-0, primers LP, RP and LBb1.3 were used to target the flanking sequences of the T-DNA. (C) Semi-quantitative RT-PCR analysis of *CIPK11* expression in Col-0 and *cipk11* plants.

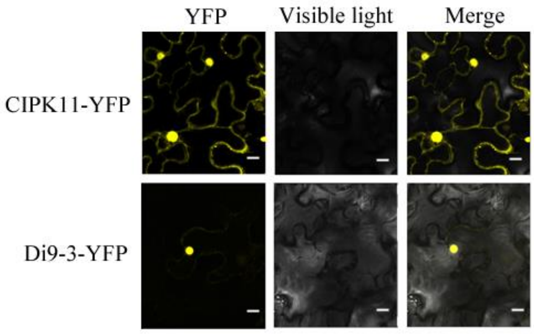

Figure S4. Subcellular localization of CIPK11 and Di19-3 proteins in the epidermal peel cells of *Nicotiana benthamiana*. All the constructs showed cytoplasmic and nucleoplasmic localization when they were transiently expressed in *Nicotiana benthamiana* epidermal cells.

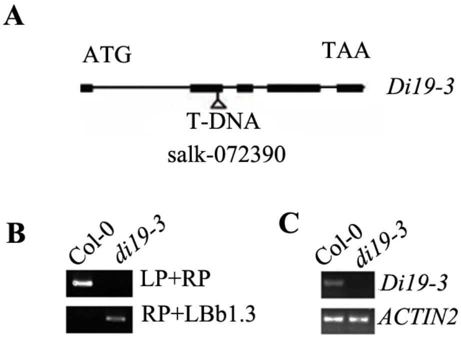

Figure S5. Identification of Arabidopsis *di19-3* mutant. (A) Structure of Arabidopsis *Di19-3* and T-DNA insertion site in *di19-3* mutant (Salk_072390). (B) Molecular analysis of *di19-3* and Col-0, primers LP, RP and LBb1.3 were used to target the flanking sequences of the T-DNA. (C) Semi-quantitative RT-PCR analysis of *Di19-3* expression in Col-0 and *di19-3* plants.

Table S1. List of primers used in the current study.

S.N.	Name of primer	Sequence of primers
**1**	SALK_108074(*cipk11*)-LP	5'-CGCGTTTAAACTCTTCACAGC-3'
**2**	SALK_108074(*cipk11*)-RP	5'-ATCTTTTTAAAAGCTTCCGCG-3'
**3**	SALK_072390(*di19-3*)-LP	5'-TCATATAACCCTTCAGCACGC-3'
**4**	SALK_072390(*di19-3*)-RP	5'-TGATTAGTTTGGGGATACCCC-3'
**5**	CIPK11-F	5'-CGGAATTCATGCCAGAGATCGAGATTGC-3'
**6**	CIPK11-R	5'-CGGGATCCAATAGCCGCGTTTGTTGACGAC-3'
**7**	LBb1.3	5'-ATTTTGCCGATTTCGGAAC-3'
**8**	ACT2 RT-F	5'-TCAGATGCCCAGAAGTCTTGTTC-3'
**9**	ACT2 RT-R	5'-GTGGATTCCAGCAGCTTCCA-3'
**10**	35S-F	5'-CAAACGAATCTCAAGCAATC-3'
**11**	FLAG-R	5'-TGTCGTCATCGTCTTTGTAGTC-3'
**12**	DI19-3-GST-F	5'-CGGGATCCATGGATTCCGATTCATGGAGT-3'
**13**	DI19-3-GST-R	5'-CGGAATTCTTATAAGCTGTCATCAAGAATCGT-3'
**14**	CIPK11-KD-GST-F	5'-CGGGATCCATGCCAGAGATCGAGATTGC-3'
**15**	CIPK11-KD-GST-R	5'-CGGAATTCTTATCCTCTAACAAACCAAGGATC-3'
**16**	pUC-SPYCE-CIPK11-F	5'-ACGGATCCATGCCAGAGATCGAGATTGC-3'
**17**	pUC-SPYCE-CIPK11-R	5'-CGGTCGACAATAGCCGCGTTTGTTGACGAC-3'
**18**	pUC-SPYCE-CIPK11-KD-F	5'-ACGGATCCATGCCAGAGATCGAGATTGC-3'
**19**	pUC-SPYCE-CIPK11-KD-R	5'-CGGTCGACTCCTCTAACAAACCAAGGATCT-3'
**20**	pUC-SPYNE-DI19-3-F	5'-CGGGATCCATGGATTCCGATTCATGGAGT-3'
**21**	pUC-SPYNE-DI19-3-R	5'-CGGAATTCTAAGCTGTCATCAAGAATCGT-3'
**22**	pGBKT7-CIPK11-F	5′-CGGGATCCGTATGCCAGAGATCGAGATTG-3'
**23**	pGBKT7-CIPK11-R	5′-ACGCGTCGACAAATAGCCGCGTTTGTTGAC-3'
**24**	pGBKT7-CIPK11-KD-F	5'-CGGGATCCATGCCAGAGATCGAGATTGC-3'
**25**	pGBKT7-CIPK11-KD-R	5'-ACGCGTCGACTTATCCTCTAACAAACCAAG-3'
**26**	pGADT7-DI19-3-F	5'-CGGGATCCATGGATTCCGATTCATGGAGT-3'
**27**	pGADT7-DI19-3-R	5'-CGGAATTCTTATAAGCTGTCATCAAGAATCGT-3'
**28**	RD29A-real time-F	5′-CAGAGGAACCACCACTCAACACA-3′
**29**	RD29A-real time-R	5'-CTCTAGGTTTACCTGTTACGCCTG-3'
**30**	RD29B-real time-F	5'-ATGGAGTCACAGTTGACACGTCCT-3'
**31**	RD29B-real time-R	5'-CTTCTGGGTCTTGCTCGTCATACT-3'
**32**	RAB18-real time-F	5'-ATGGCGTCTTACCAGAACCGTCCA-3'
**33**	RAB18-real time -R	5'-ACCACCACTTTCCTTGTGGAGTTG-3'
**34**	DREB2A-real time-F	5'-GAATGGTGCGGAAGAGATGAAG-3'
**35**	DREB2A-real time -R	5'-GTTCAAACTCGCTCAGCCAAT-3'
**36**	CIPK11-real time-F	5'-GTCGGGATTGTTTGCTGGTTGT-3'
**37**	CIPK11-real time-R	5'-TTCCCATTTTGCCCCTCCATCT-3'
**38**	DI19-3-real time-F	5'-TTGTCTCTCTCTGCTGCCACAT-3′
**39**	DI19-3-real time-R	5'-TCTTGGTTTTCTCTTGCGGTGC-3′

**40**	ACTIN2-real time-F	5'-TTGACTACGAGCAGGAGATGG-3′
**41**	ACTIN2-real time-R	5'-ACAAACGAGGGCTGGAACAAG-3′

© 2019 by the authors. Submitted for possible open access publication under the terms and conditions of the Creative Commons Attribution (CC BY) license (http://creativecommons.org/licenses/by/4.0/).
